# Pemphigus Vulgaris Unmasked in a Patient With Behçet’s Disease: A Complex Diagnostic Dilemma

**DOI:** 10.7759/cureus.70253

**Published:** 2024-09-26

**Authors:** Grissel Rios, Keysha Gonzalez-Ramos, Adriana Figueroa-Diaz, Ariana Gonzalez-Melendez

**Affiliations:** 1 Rheumatology, University of Puerto Rico, Medical Sciences Campus, San Juan, PRI; 2 Dermatology, University of Puerto Rico, Medical Sciences Campus, San Juan, PRI; 3 Rheumatology, Hospital Damas, Ponce, PRI

**Keywords:** autoimmune, behçet's disease, blistering, coexistence, pathophysiology, pemphigus vulgaris

## Abstract

Behçet's disease (BD) is a systemic vasculitis characterized by recurrent painful oral and genital ulcers, uveitis, and skin lesions. Pemphigus vulgaris (PV), on the other hand, is an autoimmune blistering disorder affecting the mucous membranes and skin, characterized by the presence of intraepidermal vesicles. Herein, we present a female in her 40s with a history of BD who presented to the emergency department with worsening oral and vaginal ulcers and extensive bullae of four months onset. A skin biopsy revealed an intraepidermal vesicle with preservation of the basal layer consistent with PV. A complete workup including vasculitides, connective tissue diseases, and human leukocyte antigen (HLA)-B*51 was performed, which revealed a positive HLA-B*51. She was treated with oral corticosteroids, rituximab, dapsone, and azathioprine. After nine months, she has remained stable. Our case suggests there may be a shared pathway in the pathophysiology of BD and PV, providing valuable insights for treatment decisions.

## Introduction

Behçet's disease (BD), also known as Behçet's syndrome, is a systemic variable vessel vasculitis characterized by recurrent painful oral and genital ulcers, uveitis, and skin lesions (folliculitis, papulopustular lesions, acneiform nodules, and erythema nodosum) [1-4]. Other manifestations include arthritis, gastrointestinal, and central nervous system involvement. The pathogenesis of BD is not well known. Still, it is proposed to be driven by genetic polymorphisms, innate and adaptive immune systems, and inciting events like infection or trauma activating the immune system and resulting in inflammation and clinical symptoms [5].

Pemphigus vulgaris (PV) is one of the subtypes of pemphigus, an autoimmune disease of skin and mucous membranes in which acantholysis results in bullae and erosion formation associated with significant morbidity [6]. The pathogenesis involves the presence of immunoglobulin (Ig) antibodies against proteins on the cell surface of keratinocytes. Antigenic targets for PV include cadherins such as desmogleins (Dsg) 1 and 3. Dsg 3 is mainly expressed in the deeper layers of the epidermis with higher expression in mucosal tissue; therefore, antibodies against Dsg 3 manifest as mucosal-predominant PV. On the other hand, antibodies against Dsg 1 present as cutaneous manifestations due to a predominance of Dsg 1 expression in the non-mucosal epidermis [6].

The possibility that BD and PV share a common pathophysiological pathway suggests a genetic connection between these conditions. Notably, individuals with one of these diseases may be more susceptible to the other due to potential overlapping mechanisms. A comprehensive understanding of the underlying pathophysiology in both disorders could hold the key to more effective treatment strategies, assisting clinicians in making informed decisions for patients affected by these complex autoimmune conditions.

## Case presentation

This is a case of a 42-year-old woman with a history of BD and a former smoker who presented to the emergency department with worsening oral and vaginal ulcers along with diffuse and extensive bullae formation on her back and abdomen for the past four months. She was diagnosed with BD four years before admission, which manifested as recurrent and persistent oral and vaginal ulcers and pathergy. During that time, she was treated with colchicine 0.6 milligram (mg) orally twice a day, methylprednisolone 8 mg twice daily, and mometasone furoate 0.1% ointment twice daily with partial improvement. Treatment was initiated with apremilast 30 mg twice daily, which was later changed to adalimumab 40 mg subcutaneously every two weeks due to recurrent urinary tract infections and eye infections while taking apremilast, although these are not typical side effects of apremilast. This change occurred 18 months before the presentation. She was on adalimumab for six months, which was discontinued one year before admission, given the inefficacy in treating the symptoms. Several months later, azathioprine 50 mg twice daily was added to the regimen because there was minimal improvement in symptoms.

Three months before admission, she developed new skin vesicles and blisters in the lower abdomen and upper back. A biopsy of the lesions showed folliculitis with neutrophilic pustules. Immunostaining was positive for the presence of varicella-zoster virus in the epidermis and negative for herpes simplex 1 and 2 or fungal organisms. She was treated with oral and intravenous (IV) acyclovir with an improvement of vesicular lesions. She then developed bullous lesions on her trunk and extremities, with worsening oral ulcers. A biopsy from the trunk revealed interface dermatitis with eosinophils compatible with herpes zoster-associated erythema multiforme.

**Figure 1:**
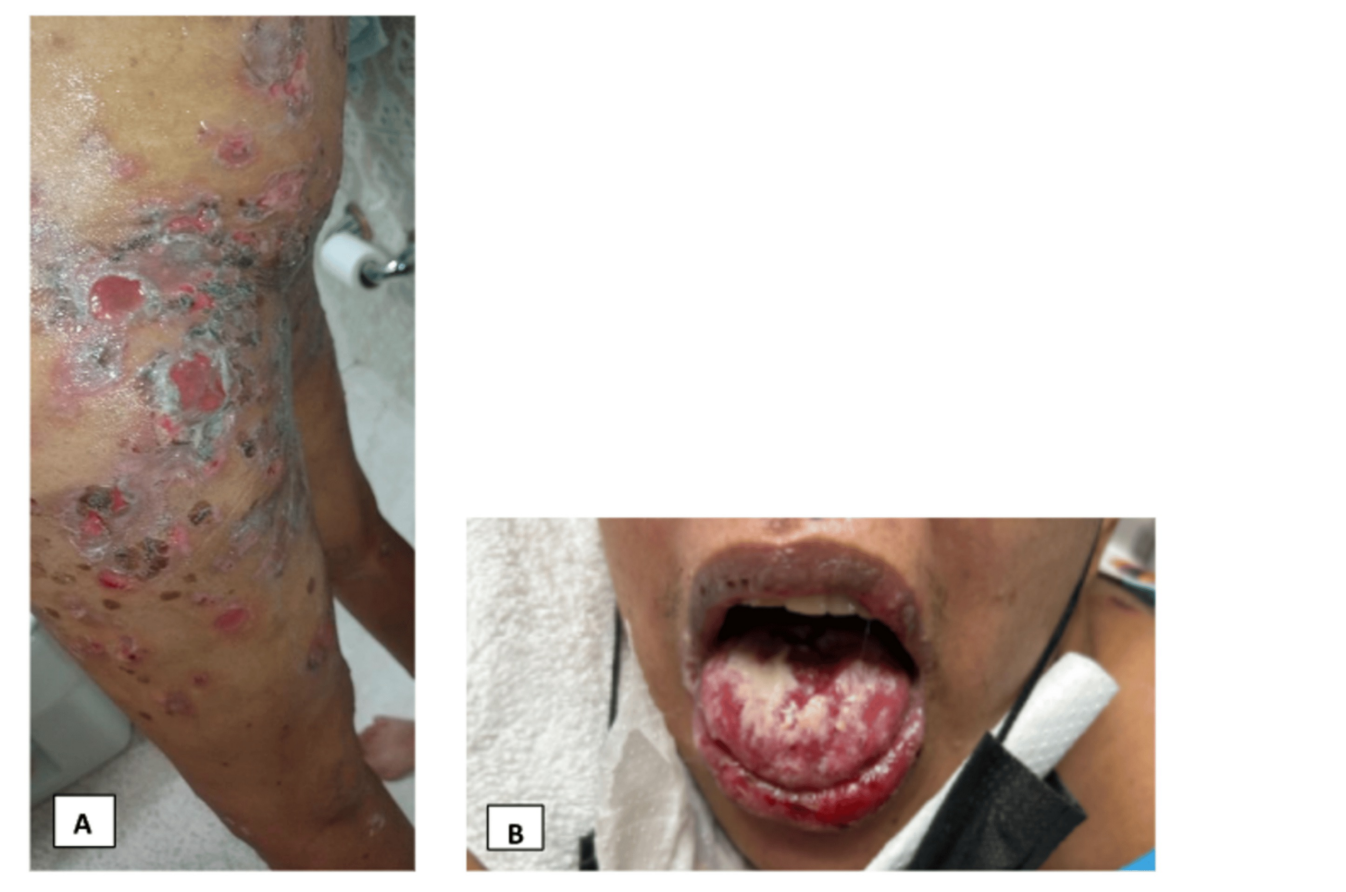
Skin lesions. (A) The left thigh exhibits eroded papules and plaques with skin sloughing and hemorrhagic crust, along with a few stage 2 ulcers on an erythematous base. (B) Mucosal ulcers and oral thrush are present, showing erosion of the lower vermilion lip with hemorrhagic crust.

Upon admission, on initial physical examination, the temperature was 36.6°C, heart rate was 120 beats per minute, and blood pressure was 122/76 millimeters of mercury. She had eroded plaques, vesicles, and bullae on the face, trunk, and bilateral upper and lower extremities. Skin sloughing was observed, involving 25% of the total body surface area (Figure 1A). The patient had ulceration on the genital and oral mucosa (Figure 1B). There was a conjunctival injection of the right eye. The musculoskeletal exam showed no synovitis on joints. She was admitted to the intensive care unit for closer monitoring.

Investigations

Laboratories show anemia, thrombocytosis, elevated inflammatory markers, elevated anti-Dsg 1 and 3 antibodies, and positive human leukocyte antigen (HLA)-B*51 (Table 1).

**Table 1:** Laboratories and serologies performed during hospitalization Dsg: desmoglein; HLA: human leukocyte antigen

Test	Result	Reference Range
White Blood Cell Count	10.69 x10^9/Liter (L)	4.0-11.0 x10^9/L
Hemoglobin	11.1 grams (g)/dL	12.1-15.1 g/dL
Platelet Count	502 x10^9/L	150-450 x10^9/L
Absolute Neutrophil Count	6,307/microliter (µL)	1,500-8,000/µL
Serum Creatinine	0.51 miligram (mg)/deciliter (dL)	0.6-1.3 mg/dL
Alkaline Phosphatase	52 mg/dL	44-121 mg/dL
Aspartate Aminotransferase	14 mg/dL	10-40 mg/dL
Alanine Aminotransferase	7 mg/dL	7-56 mg/dL
Urinalysis		
- Red Blood Cells	0-3/high power field (hpf)	0-3/hpf
- White Blood Cells	26-50/hpf	0-5/hpf
- Casts	None	None
- Proteinuria	None	None
Erythrocyte Sedimentation Rate	15 mm/hour (hr)	<20 mm/hr
C-reactive Protein	120 mg/dL	<10 mg/L
Rheumatoid Factor	11.3 units (u)/mililiter (mL)	0-18 u/mL
Antinuclear Antibodies	Negative	Negative
Antineutrophil Cytoplasmic Antibodies	Negative	Negative
Cryoglobulins	Negative	Negative
Complements (C3, C4)	C3: 105, C4: 28.5	C3: 90-180 mg/dL, C4: 10-40 mg/dL
Hepatitis B and C Tests	Negative	Negative
Human Immunodeficiency Virus	Negative	Negative
Dsg-1 Antibody	24	<14
Dsg-3 Antibody	180	<9
HLA-B*51	Positive	Negative

Differential diagnosis

**Figure 2:**
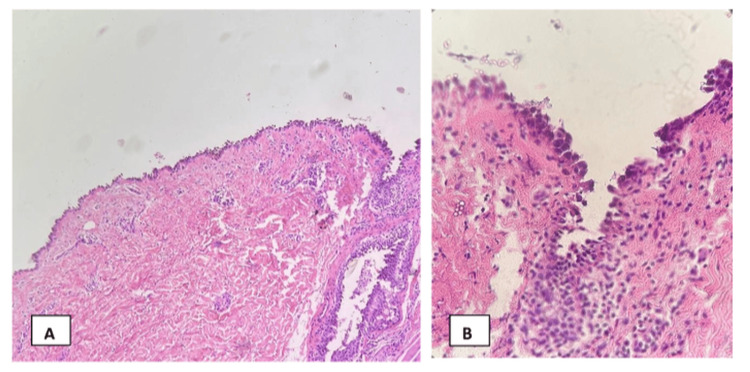
Histopathology. (A) Suprabasilar acantholysis with a preserved basal layer, exhibiting “tombstoning” (H&E stain at 20x magnification). (B) Acantholysis involving hair follicles (H&E stain at 40x magnification).

During her hospitalization, she was evaluated by an ophthalmology service, and there was no uveitis. Dermatology service evaluated the patient, and their principal differential diagnosis was PV. A punch biopsy on her left flank revealed an intraepidermal vesicle with suprabasilar acantholysis and preservation of basal layer “tombstoning” consistent with PV (Figures 2A-2B). Direct immunofluorescence of previous tissue block revealed IgG, C3, kappa, and lambda at epidermal intercellular space consistent with pemphigus not otherwise specified. Other entities including Staphylococcal scalded skin syndrome, toxic shock syndrome, toxic epidermal necrolysis, varicella zoster virus-induced pemphigus-like lesions, and tumor necrosis factor (TNF) alpha-induced PV were considered. Still, the patient was treated appropriately for varicella zoster virus and persisted with skin lesions. Also, she had not been exposed to adalimumab for one year after the symptoms presented.

Treatment

All her medications for BD, including colchicine and azathioprine, were discontinued. She received fluid resuscitation, pain management, and local wound care. She was started on IV methylprednisolone 1 mg/kilogram daily. Petroleum jelly was applied daily to skin lesions for moisture retention and barrier protection, and normal saline was used for bathing. She received oral nystatin washes for Candida infection. After receiving high-dose steroids for three days, rituximab 1000 mg IV was started and was administered on day 0 and the second dose on day 14. Oral and skin lesions significantly improved after eight weeks.

The hospital course was complicated by methicillin-sensitive *Staphylococcus aureus* bacteremia, for which she was given a course of IV cefazolin. She was discharged home on prednisone 60 mg daily, omeprazole 40 mg daily, osteoporosis prophylaxis with alendronate 70 mg weekly, and triamcinolone acetonide 0.1% ointment to apply to the trunk and extremities twice daily.

Outcome and follow-up

**Figure 3:**
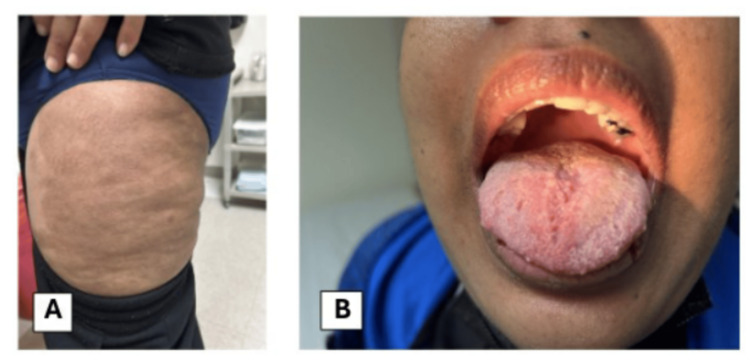
Skin lesions after treatment. (A) Diffuse patches of hyperpigmentation on the left thigh. (B) Resolution of ulceration on the tongue.

One month after discharge, mucosal and skin lesions had improved without complete resolution of mucosal symptoms. Oral dapsone 50 mg daily was started after testing for glucose-6-phosphate dehydrogenase deficiency. The prednisone dose was decreased to 50 mg, and azathioprine 50 mg daily was started. Six months after the initial reported presentation, the patient’s skin (Figure 3A) and mucosal lesions (Figure 3B) had improved significantly with resolution of vaginal ulcers. Her current treatment regimen includes dapsone 100 mg daily, azathioprine 150 mg daily, and prednisone 10 mg daily. Also, after a multidisciplinary discussion between dermatology and rheumatology, it was recommended that rituximab infusions be continued every six months.

## Discussion

We present a case of a female diagnosed with BD who subsequently developed severe PV four years later. She responded well to rituximab, azathioprine, dapsone, and prednisone, with improved mucocutaneous lesions six months later. This clinical scenario encourages further investigation through serology and HLA testing to explore the potential link between the pathophysiology of these two complex diseases. Our findings not only contribute to the limited literature on concurrent PV and BD and raise intriguing questions about shared genetic factors influencing disease processes and treatment responses but also emphasize the theory that they are genetically connected. This case emphasizes the need to recognize the rare coexistence of these two conditions and explore common therapeutic approaches.

To our knowledge, this is one of the few reported cases of simultaneous PV and BD. There was one case report of a patient who presented with nail fold skin lesions consistent with PV with negative serology and ulcers in the oral and genital mucosa along with folliculitis-like nodules and was diagnosed concomitantly with BD [7]. Another case of a patient with pemphigus foliaceus developed BD three years after remission of the pemphigus foliaceus [8]. Both entities may share a pathophysiological pathway. BD has a polygenetic predisposition. Besides HLA-B*51, susceptibility loci for BD have been identified at chemokine receptor types 1 and 3, signal transducer and activator of transcription protein family member 4, killer cell lectin-like receptor subfamily C, member 4, killer cell lectin-like receptor subfamily K, member 1, and endoplasmic reticulum aminopeptidase 1 (ERAP1) [5]. The reduced ERAP-1 activity variant reduces affinity for peptidomes due to an elevated proportion of nonamers resulting from decreased trimming activity. HLA-B*51 exhibits decreased affinity for longer peptides, like nonamers, and a reduced affinity for the Ala2 subpeptidome. These lower-affinity peptides could harm antigen presentation, potentially leading to heightened natural killer (NK) cell activation and disruptions in T-cell responses. The inflammatory process is triggered by neutrophils and sustained by gamma-delta T cells, which induce strong T helper (Th)-1, Th-2, and Th17 responses. Polymorphisms in the TNF-α gene are also implicated in BD through the nucleotide-binding domain, leucine-rich-containing family, and pyrin domain-containing-3 inflammasome pathway [9].

The NK cells play a role in BD, specifically NK1 cells, with a predominance of interferon-gamma [8]. The balance between NK1/NK2 ratio appears to mirror disease activity in BD, potentially triggering disease activity by promoting a Th1 response. This pattern has been observed in conditions like multiple sclerosis and PV [5]. Advancements in the study of T cells in pemphigus have been ongoing, focusing on T follicular helper cells, which trigger the molecular processes responsible for antibody production in B cells [10]. The role of B cells is better recognized in PV. B cells produce IgG antibodies that target proteins on the surface of keratinocytes. These proteins, known as Dsg, are transmembrane glycoproteins found in desmosomes, crucial for cell-cell adhesion in the epidermis. One systematic review of cytokine research in pemphigus revealed elevated levels of TNF-α, TGF-β, interleukin (IL)-8, IL-10, IL-12, IL-17, and IL-21, along with reduced levels of IL-2 and IL-23 [10]. The innate and adaptive pathways play a crucial part in the pathogenesis of BD and PV. These shared pathways in the pathophysiology of BD and PV assist in the treatment decision of these diseases.

The treatment of BD follows the guidelines of the European League Against Rheumatism [1]. The goal of treatment is to prevent recurrences and irreversible organ damage. Corticosteroids and colchicine were initially used for mucocutaneous involvement. TNF-α inhibitors, azathioprine, and apremilast are commonly used too. Other treatments in different studies [8,11-12] include dapsone, tocilizumab, rituximab, thalidomide, anakinra, ustekinumab, and secukinumab. The pathogenesis of BD involves T cell involvement, which has been extensively studied, but several studies have suggested a possible pathogenetic role of B cells as well [1,8]. A few case reports have described the role of rituximab in patients with BD [10]. This explains why our patients’ mucosal lesions improved with the use of rituximab.

The literature supports corticosteroids as first-line therapy for all forms of pemphigus [10]. The dose is titrated based on the severity of the disease, and the addition of a steroid-sparing agent is used for moderate-severe disease. In the past, mycophenolate mofetil or azathioprine were agents of choice. However, recent data shows the use of an anti-CD20 monoclonal antibody, rituximab, is preferred both at disease onset and in refractory disease. Azathioprine is a prodrug that, when metabolized, leads to the production of thioinosinic and thioguanine nucleotides. These acids, in turn, reduce the counts of B and T cells by inhibiting purine synthesis. Therefore, it is a medication that will help both BD and PV. Other suggested therapies for PV include cyclophosphamide, IV Ig, and dapsone [1,10]. It has been found that dapsone's anti-inflammatory effect is attributed to its capacity to inhibit the migration of neutrophils and the production of harmful secretory substances that can harm the skin, such as in PV [12]. This reinforces the pathophysiologic complexity of BD and PV and why these diseases often represent therapeutic challenges and can result in significant morbidity to patients.

The possibility of medication-induced PV was acknowledged; however, the timeframe between exposure to the medication and the onset of bullae formation did not align, leading to its exclusion. There have been studies showing the occurrence of anti-TNF-α-induced lupus-like syndrome, systemic lupus erythematosus, interstitial lung disease, new-onset or worsening of psoriasis, and cutaneous and systemic vasculitis at average intervals of 16.2-34.5 months following the onset of treatment [13]. The most common anti-TNF-α inhibitors reported have been infliximab and adalimumab. Nevertheless, cases of autoimmune blistering diseases have also been reported [14,15]. Bullous pemphigoid (BP) stands out as the most extensively documented autoimmune bullous dermatosis [13,15,16]. The time from the beginning of TNF-α inhibitor therapy to the appearance of BP varied from 1 to 209 weeks, as reported in one study [15]. Our patient had been on adalimumab for six months but had discontinued the medication for a year, which reduced the probability of PV being induced by the medication.

## Conclusions

The presented case underscores the complex interplay between BD and PV, highlighting a rare but significant clinical scenario where both conditions coexist. The patient's positive response to rituximab, azathioprine, dapsone, and prednisone, leading to improvement in mucocutaneous lesions, suggests a potential overlap in the therapeutic approaches for these diseases. This case adds to the limited literature on the simultaneous occurrence of BD and PV, raising essential questions about shared pathophysiological pathways and genetic factors. The findings advocate for further research into common underlying mechanisms and genetic predispositions that could influence the development and treatment of these autoimmune conditions.

Additionally, the treatment approaches for both BD and PV, involving corticosteroids, immunosuppressants, and targeted biologics, reflect the need for tailored therapeutic strategies that address the unique challenges posed by these conditions. Despite the complex nature of their pathogenesis, the use of medications like rituximab and dapsone demonstrates the potential for effective management through a nuanced understanding of their shared immunological pathways. This case emphasizes the importance of recognizing and addressing the coexistence of BD and PV in clinical practice, which may enhance treatment outcomes and contribute to better patient care in the future.

## References

[1] Hatemi G, Christensen R, Bang D (2018). 2018 update of the EULAR recommendations for the management of Behçet's syndrome. Ann Rheum Dis.

[2] Scherrer MA, Rocha VB, Garcia LC (2017). Behçet's disease: review with emphasis on dermatological aspects. An Bras Dermatol.

[3] Takeno M (2022). The association of Behçet's syndrome with HLA-B51 as understood in 2021. Curr Opin Rheumatol.

[4] International Team for the Revision of the International Criteria for Behçet's Disease (ITR-ICBD) (2014). The International Criteria for Behçet's Disease (ICBD): a collaborative study of 27 countries on the sensitivity and specificity of the new criteria. J Eur Acad Dermatol Venereol.

[5] van der Houwen TB, van Hagen PM, van Laar JA (2022). Immunopathogenesis of Behçet's disease and treatment modalities. Semin Arthritis Rheum.

[6] Malik AM, Tupchong S, Huang S, Are A, Hsu S, Motaparthi K (2021). An updated review of pemphigus diseases. Medicina (Kaunas).

[7] Yamaoka T, Takahashi S, Ijuin K, Nagai H, Kumagai S (2023). A case of pemphigus vulgaris with folliculitis-like nodules, genital and oral ulcers difficult to differentiate from Behçet's disease. Clin Exp Rheumatol.

[8] Caso F, Iaccarino L, Bettio S, Ometto F, Costa L, Punzi L, Doria A (2013). Refractory pemphigus foliaceus and Behçet's disease successfully treated with tocilizumab. Immunol Res.

[9] Hetta HF, Mohamed AA, Zahran AM (2021). Possible role of regulatory B cells in different Behçet’s disease phenotypes and therapies: first report from Egypt. J Inflamm Res.

[10] Takahashi H, Iriki H, Asahina Y (2023). T cell autoimmunity and immune regulation to desmoglein 3, a pemphigus autoantigen. J Dermatol.

[11] Caso F, Costa L, Rigante D (2014). Biological treatments in Behçet's disease: beyond anti-TNF therapy. Mediators Inflamm.

[12] Alkeraye S, AlZamil LR, Alenazi S (2020). Dapsone in the management of pemphigus and pemphigoid: rediscovery of its long-lost efficacy. Cureus.

[13] Wessman LL, Blixt EK, Wetter DA, Miest RY (2017). Adalimumab-associated bullous pemphigoid in a patient with ulcerative colitis. JAAD Case Rep.

[14] Boussemart L, Jacobelli S, Batteux F (2010). Autoimmune bullous skin diseases occurring under anti-tumor necrosis factor therapy: two case reports. Dermatology.

[15] Zhang J, Wang SH, Zuo YG (2022). Paradoxical phenomena of bullous pemphigoid induced and treated by identical biologics. Front Immunol.

[16] Ricci M, Zauli S, Zelante A, Trevisani L, Virgili A, Bettoli V (2014). Bullous pemphigoid occurring under anti-tumor necrosis factor-α therapy. Int J Colorectal Dis.

